# Vasoconstrictor Therapy for Acute Kidney Injury Hepatorenal Syndrome: A Meta-Analysis of Randomized Studies

**DOI:** 10.1016/j.gastha.2023.01.007

**Published:** 2023-01-18

**Authors:** Ashwani K. Singal, Geralyn Palmer, Lauren Melick, Mohamed Abdallah, Paul Kwo

**Affiliations:** 1 Department of Medicine, University of South Dakota Sanford School of Medicine, Sioux Falls, South Dakota; 2 Transplant Hepatology, Avera Transplant Institute, Sioux Falls, South Dakota; 3 Division of Gastroenterology, University of Minnesota, Minneapolis, Minnesota; 4 Division of Gastroenterology and Hepatology, Stanford University Medical Center, Stanford, California

**Keywords:** HRS, AKI, Terlipressin, Nor-epinephrine, Midodrine, Octreotide, Cirrhosis, ACLF, RRT, Liver Transplantation

## Abstract

**Background and Aims:**

Type 1 hepatorenal syndrome (HRS) is a rapid deterioration in kidney function in patients with cirrhosis. Data on efficacy of vasoconstrictors for type 1 HRS have shown mixed results.

**Methods:**

Literature searched for randomized controlled trials comparing pharmacological therapy for HRS vs placebo or another drug for HRS. Primary outcome was HRS reversal (serum creatinine <1.5mg/dL on 2 readings), and secondary outcomes were liver transplant (LT) free survival and serious adverse events (SAE).

**Results:**

Sixteen studies on 1244 patients (mean age 50.3 yrs., 67.5% males, serum creatinine of 3.07 mg/dL, serum sodium 127.2 mEq/liter, and Model for End-stage Liver Disease (MELD) score of 30.9, and Child-Pugh score 11) with type 1 HRS treated with vasoconstrictors vs placebo or another drug were analyzed. All the patients received intravenous albumin infusion. (A) terlipressin vs placebo: Odds of HRS reversal were 3.3 folds with terlipressin without difference on LT-free patient survival. Terlipressin was associated with higher odds of SAE. (B) Nor-epinephrine (NE) vs terlipressin: No difference on HRS reversal, LT-free survival, and SAE. (C) Terlipressin or NE vs midodrine and octreotide: 91% lower odds of HRS reversal with midodrine and octreotide. There were no differences on SAE (10 of 64 vs 10 of 58, *P* = .812). Non-responders vs responders had higher mean MELD score (29 vs 27.8), *P* = .014 and serum creatinine (3.5 vs 3.1), *P* = .027.

**Conclusion:**

Terlipressin and NE are similar and superior to midodrine octreotide combination for HRS reversal. No therapy improves LT-free patient survival. Response to treatment is better with lower baseline serum creatinine and MELD score. The risk of adverse effects is similar with terlipressin and NE. Studies are needed as basis to identify candidates with best response to treatment with excellent safety profile.

See editorial on page 547.

## Introduction

Renal dysfunction due to acute kidney injury (AKI) or chronic kidney disease (CKD) occurs frequently among patients with cirrhosis and end-stage liver disease and is associated with poor outcomes.^1^, ^2^, ^3^ Hepatorenal syndrome (HRS) is an etiological condition in about 20%–30% of those with renal dysfunction and is a leading cause of hospitalizations with huge economic and health care burden in these patients.^1^^,^^2^^,^^4^, ^5^, ^6^, ^7^ In a prospective study on 234 patients with cirrhosis, the incidence of HRS was 18% and 39% at 1 and 5 years follow up, respectively.^8^ HRS often presents acutely with rapid progression and high mortality (type 1 or HRS-AKI), or less commonly with an insidious onset and a more smoldering clinical course (type 2 or HRS-CKD).^1^^,^^9^

Splanchnic pooling of blood in patients with cirrhosis due to portal hypertension or due to systemic inflammation as a result of bacterial translocation leads to reduced effective blood volume and renal blood flow, putting these patients at risk of developing HRS.^1^^,^^7^^,^^10^^,^^11^ As there is no structural damage to kidneys, liver transplantation (LT) reverses the HRS and improves outcomes of these patients.^12^, ^13^, ^14^ Vasoconstrictors such as terlipressin, nor-epinephrine, midodrine, and octreotide constrict the splanchnic circulation, and when used in combination with intravenous albumin result in improved effective circulating blood volume and renal function with potential of reversal of HRS.^1^^,^^2^^,^^15^^,^^16^ Of these, terlipressin has been shown to be the most effective pharmacological agent, with improved pre- LT and post-LT patient survival among responders to terlipressin.^7^^,^^15^^,^^17^ Recently, a multicenter North American clinical trial testing terlipressin against placebo has been completed, and results of this study led to the approval of terlipressin for its use in the United States for the management of HRS.^18^ We performed this systematic review and meta-analysis of all randomized controlled trials (RCTs) examining the efficacy and safety of various vasoconstrictors in the management of HRS. As HRS-AKI is common than HRS-CKD and majority of RCTs being performed in this phenotype, we limited our study to patients with cirrhosis and HRS-AKI.

## Methods

### Literature Search

Electronic literature search was performed by the University of South Dakota librarian on Pubmed and EMBASE databases for all RCTs on HRS-AKI or type 1 HRS published between 2000 until June 2022. Mesh words used were “hepatorenal syndrome/drug therapy ”AND (“type 1” OR “type I” OR “type one”). Clinical Trial, Comparative Study, Controlled Clinical Trial, Randomized Controlled Trial were applied as filters. Manual search was also performed by the authors (GP, MA, and AKS) using cross references from studies identified on electronic search.

### Selection of Studies

**Figure 1:**
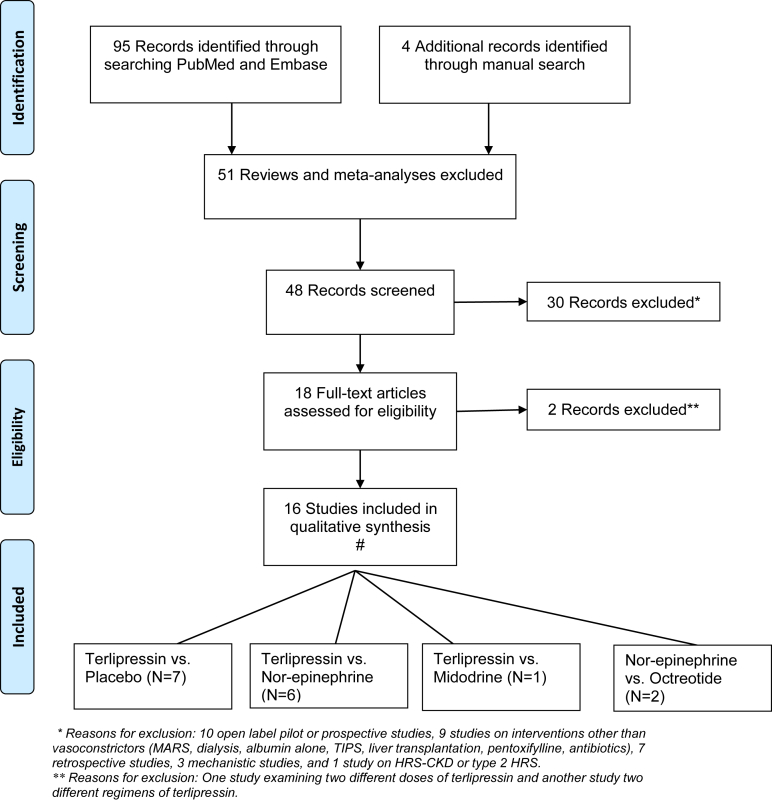
PRISMA diagram and flow chart for study selection. PRISMA, Preferred Reporting Items for Systematic Reviews and Meta-Analyses. ∗Reasons for exclusion: 10 open-label pilot or prospective studies, 9 studies on interventions other than vasoconstrictors (molecular adsorbent recirculating system, dialysis, albumin alone, transjugular intrahepatic portosystemic shunt, liver transplantation, pentoxifylline, antibiotics), 7 retrospective studies, 3 mechanistic studies, and 1 study on hepatorenal syndrome (HRS) chronic kidney disease or type 2 HRS. ∗∗Reasons for exclusion: One study examining 2 different doses of terlipressin and another study 2 different regimens of terlipressin.

From the available literature on electronic and manual search, studies were selected for analysis using the following criteria: (a) randomized study design, (b) type 1 HRS or HRS-AKI study population, (c) vasoconstrictor (terlipressin, nor-epinephrine, midodrine, and octreotide) as the intervention, (d) placebo or another vasoconstrictor as comparator, (e) reporting at least one outcome reported of HRS reversal or LT-free survival, and (f) published in English language and as full articles. All the patients irrespective of intervention arm received intravenous albumin infusion. The studies were selected by independent review of the literature by 2 authors (GP and MA), and any discrepancy was resolved by consensus among all the authors after review of the study in question. Search strategy and selection is described in detail in Figure 1.

From: Moher D, Liberati A, Tetzlaff J, Altman DG, The PRISMA Group (2009). Preferred Reporting Items for Systematic Reviews and Meta- Analyses: The PRISMA Statement. PLoS Med 6(6): e1000097. doi:10.1371/journal.pmed1000097. For more information, visit www.prisma-statement.org.

### Assessment of Study Quality

Two reviewers (MA and GP) independently assessed the methodological quality of studies using the risk of bias by Cochrane for RCTs (Figures A1 and A2).^19^^,^^20^ Assessment was based on the sequence generation for the randomization of subjects, allocation concealment, blinding of participants and personnel, blinding of outcome assessor, attrition bias, selective reporting, and other sources of bias. Trials with high or unclear risk for bias for any of the first 3 components were considered to have a high risk of bias.

### Data Extraction

Selected studies were reviewed independently by 3 authors (GP, MA, and AKS) for extracting data for each arm separately on country where the study was performed, type of intervention and comparator, sample size, patient demographics, reversal of HRS, recurrence of HRS, LT-free patient survival, serious adverse events (SAE). In addition, data were extracted on variables comparing responders vs nonresponders to treatment.

### Outcomes

The primary outcome was reversal of HRS defined as creatinine below 1.5 mg/dL. Secondary outcomes were recurrent of HRS, LT-free survival at 30-d, and SAE. If 30-day survival data were unavailable, we used LT-free survival at 14 days. As 90-day period is too far out from 30-day, we examined the 90-d LT-free survival separately.

### Statistical Analyses

Comprehensive meta-analysis software was used to analyze the data on various outcomes. Random effect model was used for pooling the data and deriving the Forest plots. Baseline characteristics were summarized as proportions for categorical and means for continuous variables. Effect size on outcomes was reported as odds ratio with 95% confidence interval. Heterogeneity across studies on the pooled data was analyzed using the I^2^ measure, defined as I^2^ >50% or *P* value < .05. For heterogeneous data, sensitivity analyses were performed to examine reasons for heterogeneity. Publication bias was assessed by visual inspection of funnel plots and Egger’s test.^21^ A minimum of 2 and 3 studies were needed for analysis on a specific outcome to report on heterogeneity and publication bias respectively. Analyses with publication bias (*P* < .1) were repeated using a nonparametric rank based Duval and Tweedie’s trim and fill test. In this test, the software repeats the analysis after excluding studies which cause skewed data, resulting in symmetric funnel plot with no significant publication bias.^22^

## Results

### Baseline Characteristics

Of the 99 studies identified on initial search, 16 RCTs examining pharmacological treatments with vasoconstrictors were included in the analysis (Figure 1), including 1244 patients with HRS-AKI randomized to intervention arm of vasoconstrictor (mean age 50.3 yrs., 67.5% males, serum creatinine of 3.07 mg/dL, serum sodium 127.2 mEq/liter, and MELD score of 30.9) and control arm with placebo or another vasoconstrictor (mean age 54.0 yrs., 67.4% males, serum creatinine of 3.11 mg/dL, serum sodium 128.4 mEq/liter, and MELD score of 30.6). Most patients were in Child-Turcott-Pugh stage C with a mean score of 10.4 in the intervention and 11 in the control arm (Table 1).

**Table 1:** Baseline Characteristics of Studies and Patients

Author, yr. ^Ref.^	Location	Intervention (N)	Regimen	Age (y.)	% M	SC	Na	MELD	CTP
		Control (N)							
Alessandria 2007^23^	Italy	Terlipressin (5)	NA	56	75	2.5	124	26	11
		Nor-epinephrine (4)		55	70	2.3	126	26	10
Arora 2020^24^	India	Terlipressin (51)	2 mg/d × 2 wk.	40.3	96	1.79	130.3	33.3	11
		Nor-epinephrine (55)	1.1 mg/h × 2 wk.	38.8	92	2.02	129.9	33.8	11.1
Boyer 2011^12^	USA, Germany	Terlipressin (29)	NA	50.6	73.2	3.1	130	33	11.7
		Placebo (35)		52.9	69.6	3.5	133	32	11.2
Boyer 2016^25^	North America	Terlipressin (97)	4 mg/d × 2 wk.	55.8	53.6	3.6	132.1	33.5	10.4
		Placebo (99)		54.8	66.7	3.7	132.1	32.6	10.3
Cavallin 2015^26^	Italy	Terlipressin (27)	NA	60	77.8	3.6	130.8	31.2	NA
		Midodrine + Octreotide (21)		65	52.3	3.8	133.5	29.1	
Goyal 2016^27^	India	Terlipressin (20)	3 mg/d × 2 wk.	54.7	85	3.35	129.7	30.1	10.9
		Nor-epinephrine (21)	13 mg/d × 2 wk.	56.9	95.2	3.14	127.9	29.2	10.8
Mahmoud 2021^28^	Egypt	Nor-epinephrine (26)	0.5–3 mg/h × 10d	59.9	60	2.72	125.2	NA	11.4
		Midodrine + Octreotide (25)	5–12.5 mg tid × 10d	61.9	40	2.47	129.9		12.1
Martin-Llahi, 2008^29^	Spain	Terlipressin (17)	NA	59	69.6	3.62	124	30	10
		Placebo (18)		55	56.5	4.12	129	28	11
Neri 2008^30^	Italy	Terlipressin (26)	1–1.5 mg/d × 2 wk.	59	38.5	2.81	126	NA	11.5
		Placebo (26)		60	42.3	2.9	126		11.2
Saif 2018^31^	India	Terlipressin (30)	3–6 mg/d × 2wk.	51.5	NA	3.2	118.5	29.1	11.9
		Nor-epinephrine (30)	1–3 mg/d × 2 wk.	53.8		3.3	119.4	30.4	12
Sanyal 2008^32^	USA, Germany	Terlipressin (56)	NA	50.6	73.2	3.96	130.6	33.4	11.7
		Placebo (56)		52.9	69.6	3.85	132.4	33.4	11.2
Sharma 2008^33^	India	Terlipressin (20)	4 mg/d × 2 wk.	48.2	85	3.0	125.2	29.6	11
		Nor-epinephrine (20)	1.5 mg/h × 2 wk.	47.8	85	3.3	124.8	31.6	10.6
Singh 2012^34^	India	Terlipressin (23)	3.1 mg/d × 2 wk.	51.6	82.6	3.27	129.3	26.4	10.7
		Nor-epinephrine (23)	0.6 mg/d × 2 wk.	48.3	82.6	3.1	128.2	24.5	10.4
Solanki, 2003^35^	India	Terlipressin (12)	NA	51.5	75	2.9	NA	NA	NA
		Placebo (12)		53.8	66.7	2.2			
Tavakkoli 2012^36^	Iran	Nor-epinephrine (6)	NA	52	63.6	2.64	118.7	32.9	11.7
		Midodrine + Octreotide (9)		52.9	66.7	2.58	121.4	34.5	11.9
Wong 2021^18^	North America	Terlipressin (199)	NA	54	60.3	3.5	133	32.7	10
		Placebo (101)		53.6	58.4	3.5	133	33.1	10.2
Summary				50.3	67.5	3.07	127.2	30.9	10.4
				54.0	67.4	3.11	128.4	30.6	11

SC, Serum creatinine; Na, Sodium; CTP, Child-Turcott-Pugh; NA, Not available.

Seven RCTs compared terlipressin vs placebo in 818 patients (454 randomized to receive terlipressin); 6 RCTs compared terlipressin vs nor-epinephrine in 312 patients (149 randomized to receive terlipressin); 2 RCTs compared nor-epinephrine vs octreotide or midodrine in 66 patients (32 randomized to receive nor-epinephrine); and 1 RCT compared terlipressin vs midodrine in 48 patients (27 randomized to receive terlipressin). Eight studies were performed in the West (4 Europe, 2 North America, and 2 multicenter studies in both Europe and United States) and 8 were reported from Asia (6 from India and 1 each from Iran and Egypt). Intravenous albumin infusion was used in both the arms of every study. Treatment was administered until HRS reversal or until a maximum of 14 days, except in the CONFIRM trial where treatment was discontinued on the day if the creatinine has not decreased by at least 30%.^18^ Dose and regimen of the vasoconstrictor therapy varied across the studies. Nine studies were of good quality with low risk of bias and remaining 7 had high risk of bias with poor study quality (Figures A1 and A2). Regarding renal replacement therapy, 2 studies included need for dialysis as failure of treatment,^18^^,^^32^ 5 studies reported data on renal replacement therapy; 56.7% of terlipressin and 80% of nor-epinephrine group,^24^ 39% of terlipressin and 50% of patients receiving placebo,^12^ 4% of patients receiving terlipressin and 5% of patients treated with midodrine and octreotide combination,^26^ 0% in patients receiving norepinephrine and 16% of patients treated with midodrine and octreotide,^36^ while none of the patients needed dialysis in the fifth study.^29^ Remaining 9 studies did not report information on renal replacement therapy.

### Outcomes

#### Terlipressin vs Placebo

##### HRS Reversal

**Figure 2:**
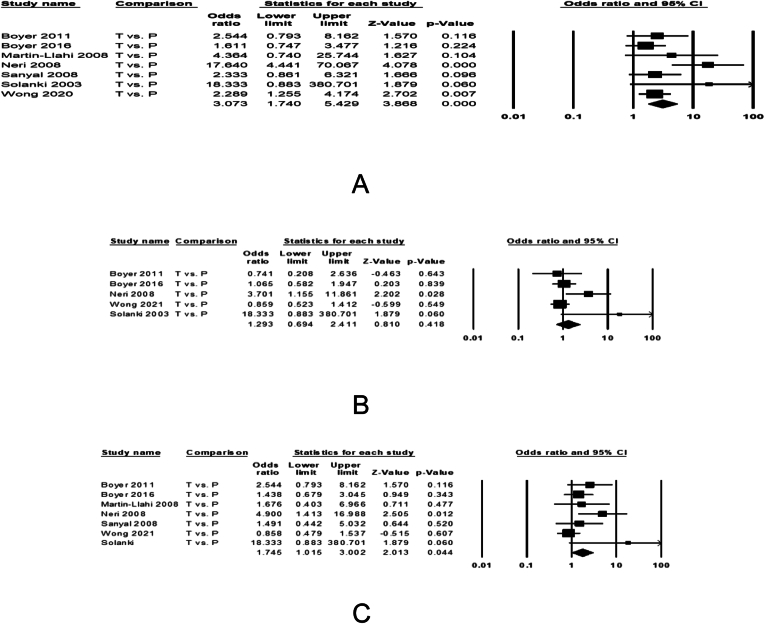
Forest plots comparing treatment with terlipressin vs placebo among patients with HRS-AKI on the pooled odds of (A) reversal of hepatorenal syndrome, (B) 30-d liver transplant free patient survival, and (C) 90-d liver transplant free patient survival. The bottom row and diamond sign represents pooled effect size with odds ratio with 95% confidence interval.

Six studies examined efficacy of terlipressin with IV albumin against IV albumin alone on reversal of HRS in 719 patients. HRS reversal was observed in 30.4% of 407 patients treated with terlipressin and 14.1% of 312 patients receiving placebo (Table A1). The pooled data showed 3.31 (1.68–6.51, *P* < .001) increased odds for HRS reversal with terlipressin (Figure 2A). The pooled data were homogeneous (I^2^ = 55, *P* = .051) without any publication bias (Egger’s *P* = .11). Sensitivity analysis after excluding the CONFIRM study, the results remain unchanged with pooled effect size of 3.62 (1.68–7.79), *P* < .001. There was trend for heterogeneity, I^2^ = 53, *P* = .058 without any publication bias, Egger’s *P* = .1.

##### Transplant-Free Survival

Data pooled from 5 studies (Boyer 2011, Boyer 2016, Neri 2008, Wong 2021, Solanki 2003) examining transplant-free survival at 30 days from the initiation of treatment in 581 patients. Of the 349 patients treated with terlipressin, 217 (62.2%) survived until 30 days compared to 150 of 232 (64.7%) receiving placebo, with similar survival between the 2 treatments, 1.29 (0.69–2.41, *P* = .418), Figure 2B. The pooled data were homogeneous (I^2^ = 55, *P* = .066) with no publication bias (Egger’s *P* = .152). Sensitivity analysis after excluding CONFIRM study, the data remained unchanged with pooled effect size 1.72 (0.67–4.40) *P* = .262, with a trend on heterogeneity, I^2^ = 58, *P* = .066 and no publication bias, Egger’s *P* = .326. Data pooled from 7 studies examining transplant-free survival at 90 days from the initiation of treatment in 652 patients. Of 363 patients treated with terlipressin, 116 (32%) survived until 90 days compared to 64 of 280 (22.1%) patients receiving placebo (Figure 2C), with 1.75 (1.02–3.00, *P* = .04) folds better survival with terlipressin. The pooled data were homogeneous (I^2^ = 42, *P* = .11) with publication bias (Egger’s *P* = .02). Sensitivity analysis after excluding CONFIRM study, the data remained unchanged with pooled effect size 2.08 (1.27–3.40) *P* = .003, with no, I^2^ = 4, *P* = .39 or publication bias, Egger’s *P* = .1.

##### Serious Adverse Effects

Terlipressin vs placebo had 2.4 folds higher risk for any SAE. Specifically, the risk was 6.2, 4.4, and 2.7 folds higher for cardiovascular, gastrointestinal, and respiratory SAE. Although, the risk of ischemia of small bowel and skin was also higher with terlipressin, these were not significant probably due to small sample size for these analyses (Table 2). Data were homogeneous without any publication bias.

**Table 2:** Pooled Data of Studies Comparing Terlipressin vs Placebo for SAE

SAE	No. of studies	N (T vs P)	Odds ratio (95% CI)	I^2^ P	Egger’s P
Overall	6	719 (407 vs 312)	2.40 (1.61–3.58)	15, 0.52	0.001
Cardiovascular	4	228 (106 vs 112)	6.2 (1.5–25.0)	0, 0.90	0.029
Gastrointestinal	4	577 (339 vs 238)	4.4 (2.0–9.8)	0, 0.97	0.075
Respiratory	5	690 (390 vs 300)	2.7 (1.3–5.7)	0, 0.90	0.21
SB ischemia	3	531 (313 vs 218)	4.8 (0.8–27.5)	0, 0.87	0.50
Skin ischemia	2	243 (118 vs 125)	5.8 (0.7–51.0)	0, 0.62	NA

T, Terlipressin; P, Placebo; CI, Confidence interval.

#### Terlipressin vs Nor-Epinephrine

##### HRS Reversal

**Figure 3:**
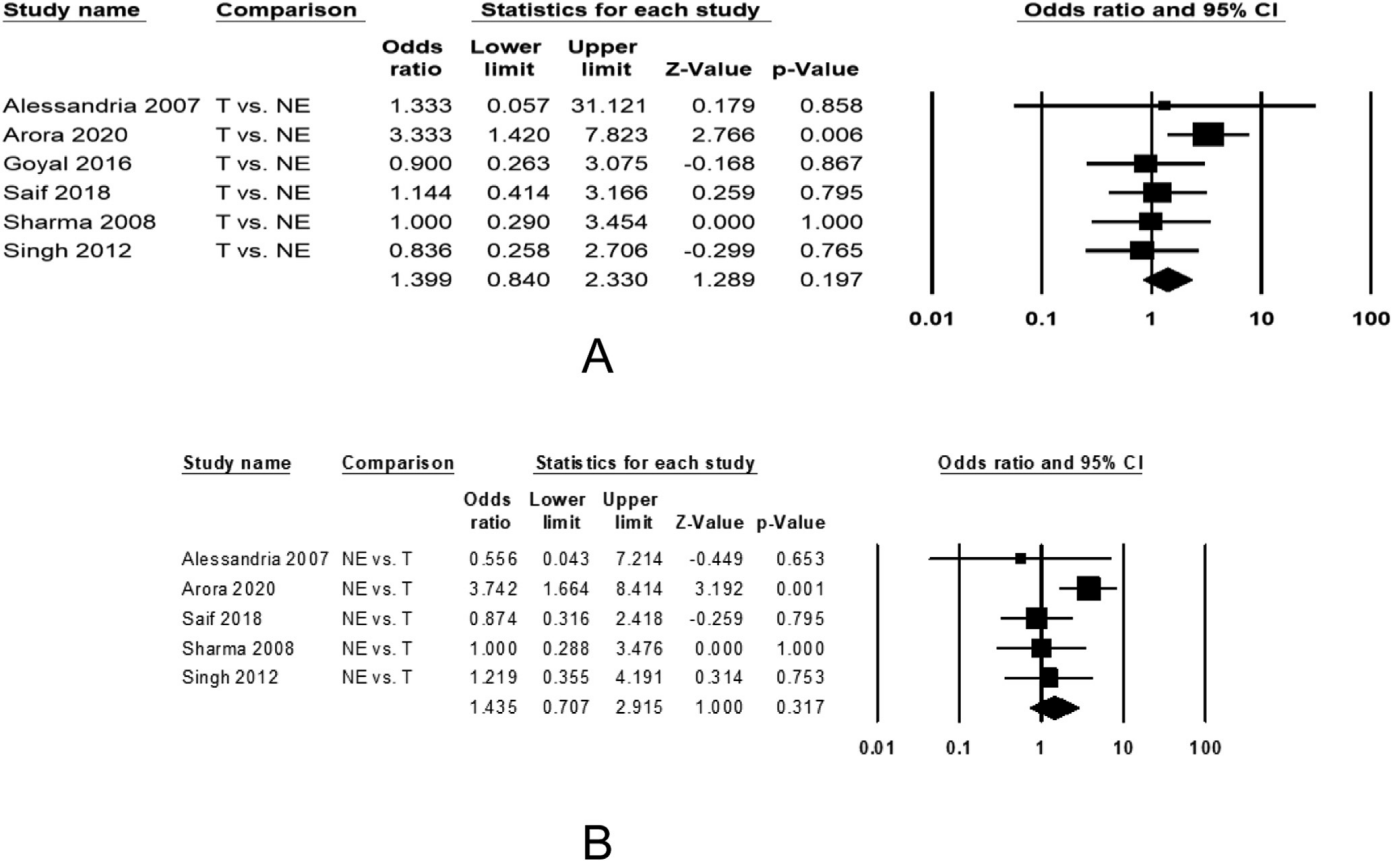
Forest plots comparing treatment with terlipressin vs nor-epinephrine among patients with HRS-AKI on the pooled odds of (A) reversal of hepatorenal syndrome and (B) 30-d liver transplant free patient survival. The bottom row and diamond sign represents pooled effect size with odds ratio with 95% confidence interval.

Six studies examined efficacy of terlipressin with IV albumin against nor-epinephrine with IV albumin on reversal of HRS in 316 patients. HRS reversal was observed in 46.2% of 158 patients treated with terlipressin and 37.3% of 156 patients treated with nor-epinephrine (Table A1). The pooled data showed no difference between the 2 drugs, 1.44 (0.88–2.36, *P* = .15) on HRS reversal (Figure 3A). The pooled data were homogeneous (I^2^ = 7, *P* = .37) without any publication bias (Egger’s *P* = .31).

##### Transplant Free Survival

Five studies examined transplant-free survival at 30 days from the initiation of treatment in 288 patients. Of 145 patients treated with terlipressin, 74 (51%) survived until 30 days compared to 56 of 143 (39.2%) receiving nor-epinephrine (Table A1), with similar survival 1.44 (0.71–2.92, *P* = .32) between the 2 arms (Figure 3B). The pooled data were homogeneous (I^2^ = 43, *P* = .14) without any publication bias (Egger’s *P* = .21). Data were not pooled for 90-day survival analysis as only one study reported this outcome.^31^

##### Serious Adverse Effects

Three studies comparing terlipressin vs nor-epinephrine reported data on SAE. Pooled data showed no diference comparing SAE between the 2 drugs, odds ratio (95% confidence interval): 1.03 (0.31–3.46), *P* = .96. Data were homogeneous (I^2^ = 32, *P* = .18) without any publication bias (Egger’s *P* = .35). There were not enough studies to examine SAE for individual organ systems, and hence these analyses were not performed for studies comparing terlipressin vs nor-epinephrine.

#### Midodrine and Octreotide

##### HRS Reversal

One study compared combination of midodrine and octreotide with IV albumin against terlipressin on 48 patients, 27 receiving terlipressin, ^26^ and 2 studies against nor-epinephrine in 74 patients, 37 treated with nor-epinephrine.^28^^,^^36^ Pooled data showed that midodrine and octreotide combination is inferior to terlipressin or nor-epinephrine, with 6.8 fold higher odds of HRS reversal with terlipressin or nor-epinephrine compared to that with midodrine and octreotide, 6.79 (2.38–19.32), *P* < .001 (Figure A3A). The pooled data were homogeneous (I^2^ = 5.7, *P* = .35) without any publication bias, Egger’s *P* = .91.

##### Transplant-Free Survival

Transplant-free survival at 30 days from the initiation of treatment was 56.7% in 53 patients who received terlipressin or nor-epinephrine and 43.5% of 46 patients receiving midodrine octreotide combination, with no difference in the pooled data 1.73 (0.73–4.09, *P* = .21) between the 2 arms in the pooled data (Figure A3B). Transplant free survival at 90 days from 2 studies was 56.3% in 32 patients who received terlipressin or nor-epinephrine and 51.9% of 27 patients receiving a combination of midodrine and octreotide, with no difference in the pooled data 1.15 (0.27–4.94, *P* = .85) between the 2 arms in the pooled data (Figure A3C). The pooled data were homogeneous (I^2^ = 38, *P* = .20). Publication bias could not be assessed with only 2 studies in the analysis.

##### Serious Adverse Effects

Among the 3 studies, 10 SAE among 64 patients receiving terlipressin *or* nor-epinephrine occurred vs 10 among 58 receiving midodrine and octreotide combination, *P* = .812. Data were homogeneous (I^2^ = 21, *P* = .32) without any publication bias (Egger’s *P* = .45). For this analysis also, there were not enough studies to examine SAE for individual organ systems.

### Predictors of Response to Treatment

**Figure 4:**
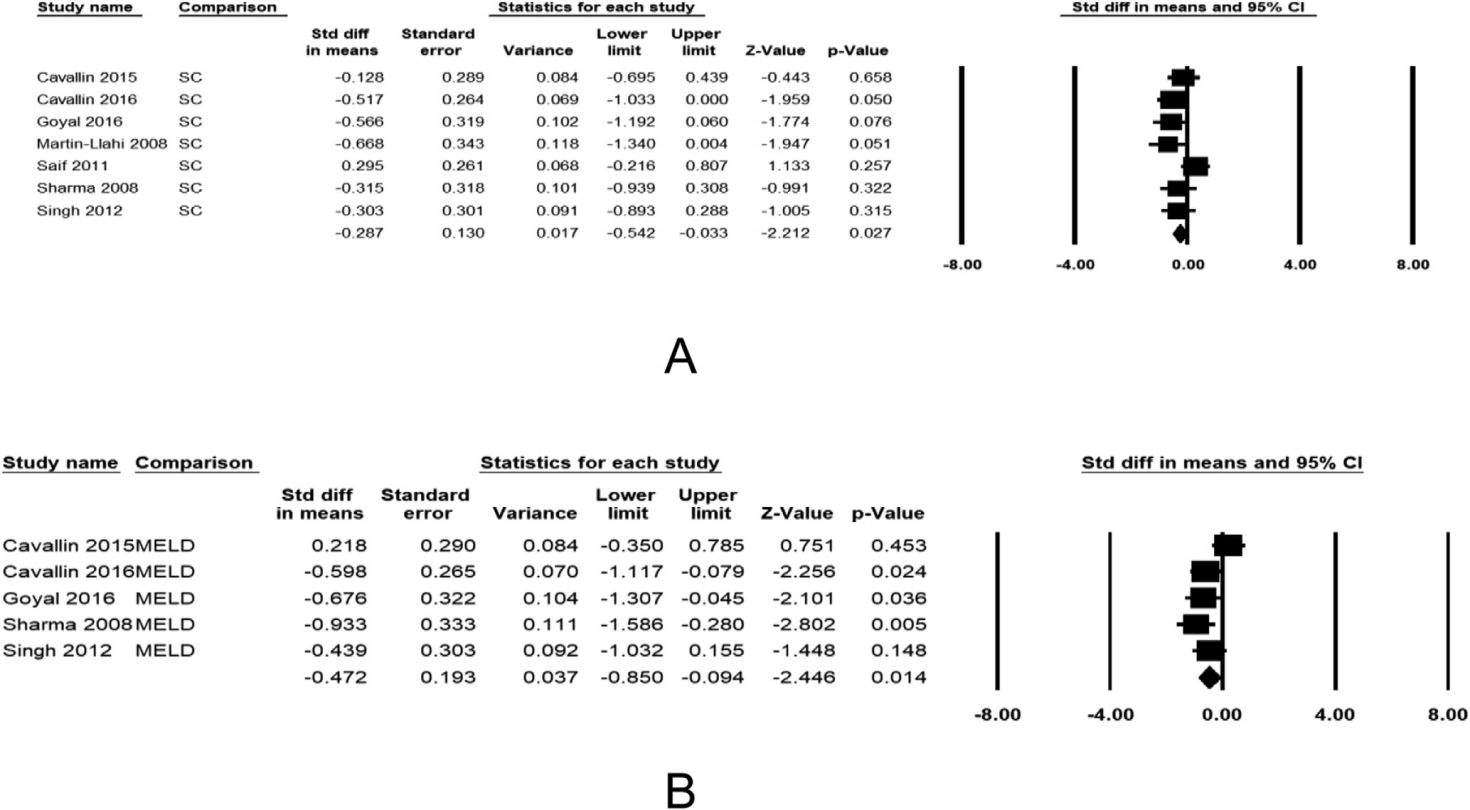
Forest plots comparing responders vs non-responders to treatment with vasoconstrictor therapy for (A) baseline serum creatinine and (B) baseline MELD score. The bottom row represents pooled mean difference between responders and non-responders with 95% confidence interval.

Seven studies were examined for baseline patient variables predicting response to the treatment. Nonresponders vs responders had higher mean MELD score (29 vs 27.8, pooled difference −0.47 [−0.85 to −0.09], *P* = .014 and serum creatinine (3.5 vs 3.1, pooled difference −0.29 [−0.54 to −0.03], *P* = .027 (Figure 4A and B). Age of the patient, baseline serum albumin prior to receiving treatment and albumin infusion, and baseline serum sodium did not predict response to the treatment (Table A2).

## Discussion

The main findings of our meta-analysis on use of vasoconstrictors with intravenous albumin in the treatment of HRS-AKI are that (a) terlipressin and nor-epinephrine are similar and superior to midodrine and octreotide for reversal of HRS, (b) higher serum creatinine and MELD score at baseline are associated with lower odds of HRS reversal, and (c) these drugs especially terlipressin and norepinephrine should be used judiciously with close monitoring for cardiopulmonary or ischemic adverse events.

Until the Food and Drug Administration (FDA) approval of terlipressin 9-14-2022 for its use in the United States, nor-epinephrine or midodrine and octreotide combination are the only available options for the management of HRS-AKI. As nor-epinephrine use in most centers and hospitals is restricted for use on patients in the intensive care units, combination of midodrine and octreotide remains the only available choice for treatment of HRS-AKI for patients managed on the medical floors. Nor-epinephrine remains the only available option among nonresponders to midodrine and octreotide, with reversal of HRS in up to 40% of these patients. In a recent study, use of nor-epinephrine on medical floors in nonresponders to midodrine and octreotide was effective in 40% of patients for HRS reversal.^16^

The response to vasoconstrictors is variable across studies with the same drug. For example, HRS reversal (serum creatinine reduction to </ = 1.5 mg/dL) was observed in 20%–80% across 14 studies. Similarly, reversal of HRS with use of nor-epinephrine across 7 studies was reported in 17%–83% of patients. Baseline renal function and liver disease severity as measured by serum creatinine level and MELD score respectively determine response to vasoconstrictors. Of these, renal function seems more clinically relevant given that the MELD score was lower by only 1.2 points in responders compared to nonresponders. Hence, patients with AKI should be identified and managed earlier for potential reversibility, and identify those with HRS, so that vasoconstrictors can be initiated sooner to improve chances of response to these medications.^1^^,^^7^^,^^37^

Improvement in renal function and reversal of HRS with terlipressin translated into improvement in LT-free survival, and no difference when compared to nor-epinephrine. It should be noted that recurrence of HRS although not examined in this study has been reported to occur in about 20% of cases. In a post hoc analysis of a randomized controlled trial on 99 patients with HRS comparing terlipressin and placebo for HRS-AKI, patient survival at 6 months was 97% in 17 selected to receive LT vs 47% in 35 patients with HRS reversal but not receiving LT vs 4% in 47 patients who did not reverse HRS and also were not selected to receive LT.^12^

The benefit of terlipressin has been consistently demonstrated including the largest randomized placebo controlled multicenter North American study (CONFIRM) on 300 patients with HRS-AKI (199 receiving terlipressin), with reversal of HRS in 32% vs 17%, *P* = .006.^18^ Approval of terlipressin by the FDA for the management of HRS in the United States is a good news for hepatologists and medical community in general; however, this approval comes with certain stipulations due to concern for safety issues including death due to respiratory failure in 22 patients (11%) within 90 days of receiving terlipressin.^18^ Increase in effective circulating blood volume with vasoconstrictors and oncotic pressure with intravenous albumin results in increased intravascular volume, potentially resulting in fluid overload with risk of respiratory failure.^7^ A post hoc analysis of the study data showed that respiratory failure events were associated with severity of HRS with serum creatinine levels and injudicious use of intravenous albumin.

Comparison of pharmacological therapies on a homogeneous group of patients with HRS-AKI or type-1 HRS including the largest study (CONFIRM) is the strength of our study compared to similar meta-analyses on this subject.^15^^,^^38^, ^39^, ^40^ However, we do recognize heterogeneous data as a limitation of our study. Differences in study population and follow-up period across studies could have resulted in heterogeneity of the pooled data. To overcome the follow-up period across studies, the outcomes were homogenized with HRS reversal as creatinine level of </ = 1.5 mg/dL and transplant-free patient survival examined at 30 or 90 days. Baseline renal and liver function probably accounted for the heterogeneity across studies, as these were the best predictors of response to treatment. Unavailable data on changes in mean arterial pressure in response to vasoconstrictor therapy probably limited the assessment of this variable on the pooled effect size. Furthermore, serum creatinine is not the perfect surrogate for true renal function; however, given advantages of serum creatinine measurement over measuring creatinine clearance, serum creatinine was used across the studies to homogenously define HRS reversal. Another limitation is the availability of only 2 studies comparing the combination of midodrine and octreotide with nor-epinephrine in one and terlipressin in another study.^26^^,^^36^

## Conclusion

In conclusion, terlipressin and nor-epinephrine are similar and superior to midodrine and octreotide in reversal of HRS. Treatment should be initiated earlier than latter given poorer response with increasing serum creatinine. Although, terlipressin is now FDA approved for use in the United States, the drug should be used cautiously in elderly with compromised or borderline cardiopulmonary function, and avoided in those with active ischemia (coronary, cerebral, gastrointestinal, and peripheral arterial), serum creatinine >5 mg/dL, MELD >35, or patients with grade 3 acute-on-chronic liver failure studies. Further, its benefits should be weighed against potential risks in those candidates who are awaiting LT. Clearly, irrespective of response to vasoconstrictor therapy, LT should be considered to improve long-term outcomes of these patients. Finally, maintenance vasoconstrictor regimens and newer therapies to improve hepatic function are needed to improve long-term outcomes,^41^ especially HRS patients ineligible for LT.

## Supplementary Materials

Tables A1–A2 and Figures A1–A3

## References

[1] Biggins S.W., Angeli P., Garcia-Tsao G. (2021). Diagnosis, evaluation, and management of ascites, spontaneous bacterial peritonitis and hepatorenal syndrome: 2021 practice guidance by the American association for the study of liver diseases. Hepatology.

[2] Russ K.B., Stevens T.M., Singal A.K. (2015). Acute kidney injury in patients with cirrhosis. J Clin Transl Hepatol.

[3] D'Amico G., Garcia-Tsao G., Pagliaro L. (2006). Natural history and prognostic indicators of survival in cirrhosis: a systematic review of 118 studies. J Hepatol.

[4] Allegretti A.S., Ortiz G., Wenger J. (2015). Prognosis of acute kidney injury and hepatorenal syndrome in patients with cirrhosis: a prospective cohort study. Int J Nephrol.

[5] Jamil K., Huang X., Lovelace B. (2019). The burden of illness of hepatorenal syndrome (HRS) in the United States: a retrospective analysis of electronic health records. J Med Econ.

[6] Singal A.K., Jackson B., Pereira G.B. (2018). Biomarkers of renal injury in cirrhosis: association with acute kidney injury and recovery after liver transplantation. Nephron.

[7] Gines P., Sola E., Angeli P. (2018). Hepatorenal syndrome. Nat Rev Dis Primers.

[8] Gines A., Escorsell A., Gines P. (1993). Incidence, predictive factors, and prognosis of the hepatorenal syndrome in cirrhosis with ascites. Gastroenterology.

[9] Angeli P., Garcia-Tsao G., Nadim M.K. (2019). News in pathophysiology, definition and classification of hepatorenal syndrome: a step beyond the International Club of Ascites (ICA) consensus document. J Hepatol.

[10] Arroyo V., Moreau R., Jalan R. (2020). Acute-on-Chronic liver failure. N Engl J Med.

[11] Huelin P., Sola E., Elia C. (2019). Neutrophil gelatinase-associated Lipocalin for assessment of acute kidney injury in cirrhosis: a prospective study. Hepatology.

[12] Boyer T.D., Sanyal A.J., Garcia-Tsao G. (2011). Impact of liver transplantation on the survival of patients treated for hepatorenal syndrome type 1. Liver Transpl.

[13] Singal A.K., Hasanin M., Kaif M. (2018). MELD stratified outcomes among recipients with Diabetes or hypertension: simultaneous liver kidney versus liver alone. J Clin Gastroenterol.

[14] Hmoud B., Kuo Y.F., Wiesner R.H. (2015). Outcomes of liver transplantation alone after listing for simultaneous kidney: comparison to simultaneous liver kidney transplantation. Transplantation.

[15] Nanda A., Reddy R., Safraz H. (2018). Pharmacological therapies for hepatorenal syndrome: a systematic review and meta-analysis. J Clin Gastroenterol.

[16] Kwong A., Kim W.R., Kwo P.Y. (2021). Feasibility and effectiveness of norepinephrine outside the intensive care setting for treatment of hepatorenal syndrome. Liver Transpl.

[17] Piano S., Gambino C., Vettore E. (2021). Response to terlipressin and albumin is associated with improved liver transplant outcomes in patients with hepatorenal syndrome. Hepatology.

[18] Wong F., Pappas S.C., Curry M.P. (2021). Terlipressin plus albumin for the treatment of type 1 hepatorenal syndrome. N Engl J Med.

[19] Higgins J.P., Altman D.G., Gotzsche P.C. (2011). The Cochrane Collaboration's tool for assessing risk of bias in randomised trials. BMJ.

[20] McGuinness L.A., Higgins J.P.T. (2021). Risk-of-bias VISualization (robvis): an R package and Shiny web app for visualizing risk-of-bias assessments. Res Synth Methods.

[21] Egger M., Davey Smith G., Schneider M. (1997). Bias in meta-analysis detected by a simple, graphical test. BMJ.

[22] Duval S., Tweedie R. (2000). Trim and fill: a simple funnel-plot-based method of testing and adjusting for publication bias in meta-analysis. Biometrics.

[23] Alessandria C., Ottobrelli A., Debernardi-Venon W. (2007). Noradrenalin vs terlipressin in patients with hepatorenal syndrome: a prospective, randomized, unblinded, pilot study. J Hepatol.

[24] Arora V., Maiwall R., Rajan V. (2020). Terlipressin is superior to noradrenaline in the management of acute kidney injury in acute on chronic liver failure. Hepatology.

[25] Boyer T.D., Sanyal A.J., Wong F. (2016). Terlipressin plus albumin is more effective than albumin alone in improving renal function in patients with cirrhosis and hepatorenal syndrome type 1. Gastroenterology.

[26] Cavallin M., Kamath P.S., Merli M. (2015). Terlipressin plus albumin versus midodrine and octreotide plus albumin in the treatment of hepatorenal syndrome: a randomized trial. Hepatology.

[27] Goyal O., Sidhu S.S., Sehgal N. (2016). Noradrenaline is as effective as terlipressin in hepatorenal syndrome type 1: a prospective, randomized trial. J Assoc Physicians India.

[28] El-Desoki Mahmoud E.I., Abdelaziz D.H., Abd-Elsalam S. (2021). Norepinephrine is more effective than midodrine/octreotide in patients with hepatorenal syndrome-acute kidney injury: a randomized controlled trial. Front Pharmacol.

[29] Martin-Llahi M., Pepin M.N., Guevara M. (2008). Terlipressin and albumin vs albumin in patients with cirrhosis and hepatorenal syndrome: a randomized study. Gastroenterology.

[30] Neri S., Pulvirenti D., Malaguarnera M. (2008). Terlipressin and albumin in patients with cirrhosis and type I hepatorenal syndrome. Dig Dis Sci.

[31] Saif R.U., Dar H.A., Sofi S.M. (2018). Noradrenaline versus terlipressin in the management of type 1 hepatorenal syndrome: a randomized controlled study. Indian J Gastroenterol.

[32] Sanyal A.J., Boyer T., Garcia-Tsao G. (2008). A randomized, prospective, double-blind, placebo-controlled trial of terlipressin for type 1 hepatorenal syndrome. Gastroenterology.

[33] Sharma P., Kumar A., Shrama B.C. (2008). An open label, pilot, randomized controlled trial of noradrenaline versus terlipressin in the treatment of type 1 hepatorenal syndrome and predictors of response. Am J Gastroenterol.

[34] Singh V., Ghosh S., Singh B. (2012). Noradrenaline vs. terlipressin in the treatment of hepatorenal syndrome: a randomized study. J Hepatol.

[35] Solanki P., Chawla A., Garg R. (2003). Beneficial effects of terlipressin in hepatorenal syndrome: a prospective, randomized placebo-controlled clinical trial. J Gastroenterol Hepatol.

[36] Tavakkoli H., Yazdanpanah K., Mansourian M. (2012). Noradrenalin versus the combination of midodrine and octreotide in patients with hepatorenal syndrome: randomized clinical trial. Int J Prev Med.

[37] Tariq R., Singal A.K. (2020). Management of hepatorenal syndrome: a review. J Clin Transl Hepatol.

[38] Gifford F.J., Morling J.R., Fallowfield J.A. (2017). Systematic review with meta-analysis: vasoactive drugs for the treatment of hepatorenal syndrome type 1. Aliment Pharmacol Ther.

[39] Thomson M.J., Taylor A., Sharma P. (2020). Limited progress in hepatorenal syndrome (HRS) reversal and survival 2002-2018: a systematic review and meta-analysis. Dig Dis Sci.

[40] Best L.M., Freeman S.C., Sutton A.J. (2019). Treatment for hepatorenal syndrome in people with decompensated liver cirrhosis: a network meta-analysis. Cochrane Database Syst Rev.

[41] Robertson M., Majumdar A., Garrett K. (2014). Continuous outpatient terlipressin infusion for hepatorenal syndrome as a bridge to successful liver transplantation. Hepatology.

