# Supplementary material for: Vasoconstrictor Therapy for Acute Kidney Injury Hepatorenal Syndrome: A Meta-Analysis of Randomized Studies
Source: Gastro Hep Adv. 2023 Jan 18;2(4):455–64. doi: 10.1016/j.gastha.2023.01.007 (PMC11308464; doi:10.1016/j.gastha.2023.01.007)
Supplement: Tables A1–A2 and Figures A1–A3 [file mmc1.docx]

**Supplementary Table 1** Outcomes on reversal of hepatorenal syndrome (serum creatinine </=1.5 mg/dL) and transplant free patient survival

| Author, Yr. ^Ref.^ | Location | Intervention (N) Control (N) | Regimen | HRS reversal (N) | 30-d survival (N) | 90-d survival (N) |
| --- | --- | --- | --- | --- | --- | --- |
| Alessandria 2007^31^ | Italy | Terlipressin (5) Nor-epinephrine (4) | NA | 4  3 | 1  1 | NA |
| Arora 2020 ^32^ | India | Terlipressin (51) Nor-epinephrine (55) | 2 mg/d x 2 wk.  1.1 mg/hr. x 2 wk. | 24  10 | 29  12 | NA |
| Boyer 2011^12^ | USA, Germany | Terlipressin (29) Placebo (35) | NA | 10  6 | 10  6 | 4  2 |
| Boyer 2016^33^ | North America | Terlipressin (97) Placebo (99) | 4 mg/d x 2 wk. | 19  13 | 22  17 | 53  52 |
| Cavallin 2015^23^ | Italy | Terlipressin (27) Midodrine (21) | NA | 15  1 | 19  14 | 16  9 |
| Goyal 2016 ^34^ | India | Terlipressin (20) Nor-epinephrine (21) | 3 mg/d x 2 wk.  13 mg/d x 2 wk. | 9  10 | NA | NA |
| Mahmoud 2021 ^25^ | Egypt | Nor-epinephrine (26)  Midodrine (25) | 0.5-3 mg/h x10d  5-12.5 mg tid x 10d | 15  5 | 11  6 | NA |
| Martin-Llahi, 2008 ^35^ | Spain | Terlipressin (17) Placebo (18) | NA | 6  2 | NA | 6  4 |
| Neri 2008 ^36^ | Italy | Terlipressin (26) Placebo (26) | 1-1.5 mg/d x 2 wk. | 21  5 | 19  11 | 14  5 |
| Saif 2018 ^37^ | India | Terlipressin (30) Nor-epinephrine (30) | 3-6 mg/d x 2wk.  1-3 mg/d x 2 wk. | 17  16 | 16  17 | 1  6 |
| Sanyal 2008 ^38^ | USA, Germany | Terlipressin (56) Placebo (56) | NA | 14  7 | NA | 7  5 |
| Sharma 2008 ^39^ | India | Terlipressin (20) Nor-epinephrine (20) | 4 mg/d x 2 wk.  1.5 mg/hr. x 2 wk. | 10  10 | 11  11 | NA |
| Singh 2012 ^40^ | India | Terlipressin (23)  Nor-epinephrine (23) | 3.1 mg/d x 2 wk.  0.6 mg/d x 2 wk. | 9  10 | 8  7 | NA |
| Solanki, 2003 ^41^ | India | Terlipressin (12) Placebo (12) | NA | 5  0 | NA | NA |
| Tavakkoli 2012 ^24^ | Iran | Nor-epinephrine (6) Octreotide (9) | NA | 5  6 | NA | 2  5 |
| Wong 2021 ^18^ | North America | Terlipressin (199) Placebo (101) | NA | 63  17 | 121  65 | 52  27 |

*NA: Not available*

**Supplementary Table 2** Predictors of response to vasoconstrictors in acute kidney injury hepatorenal syndrome

|  | Std. diff in means | 95% CI | P value | I^2^, P | Egger’s P |
| --- | --- | --- | --- | --- | --- |
| Age in years | -0.13 | -0.39 to 0.13 | 0.32 | 0, 0.52 | 0.38 |
| SC in mg/Dl | -0.29 | -0.54 to -0.03 | 0.027 | 25, 0.24 | 0.15 |
| Meld Score | -0.47 | -0.85 to -0.09 | 0.014 | 51, 0.08 | 0.53 |
| SA in gm/dL | -0.22 | -0.50 to 0.07 | 0.13 | 0, 0.84 | 0.58 |

*SC: Serum creatinine; SA: Serum albumin; CI: Confidence interval*


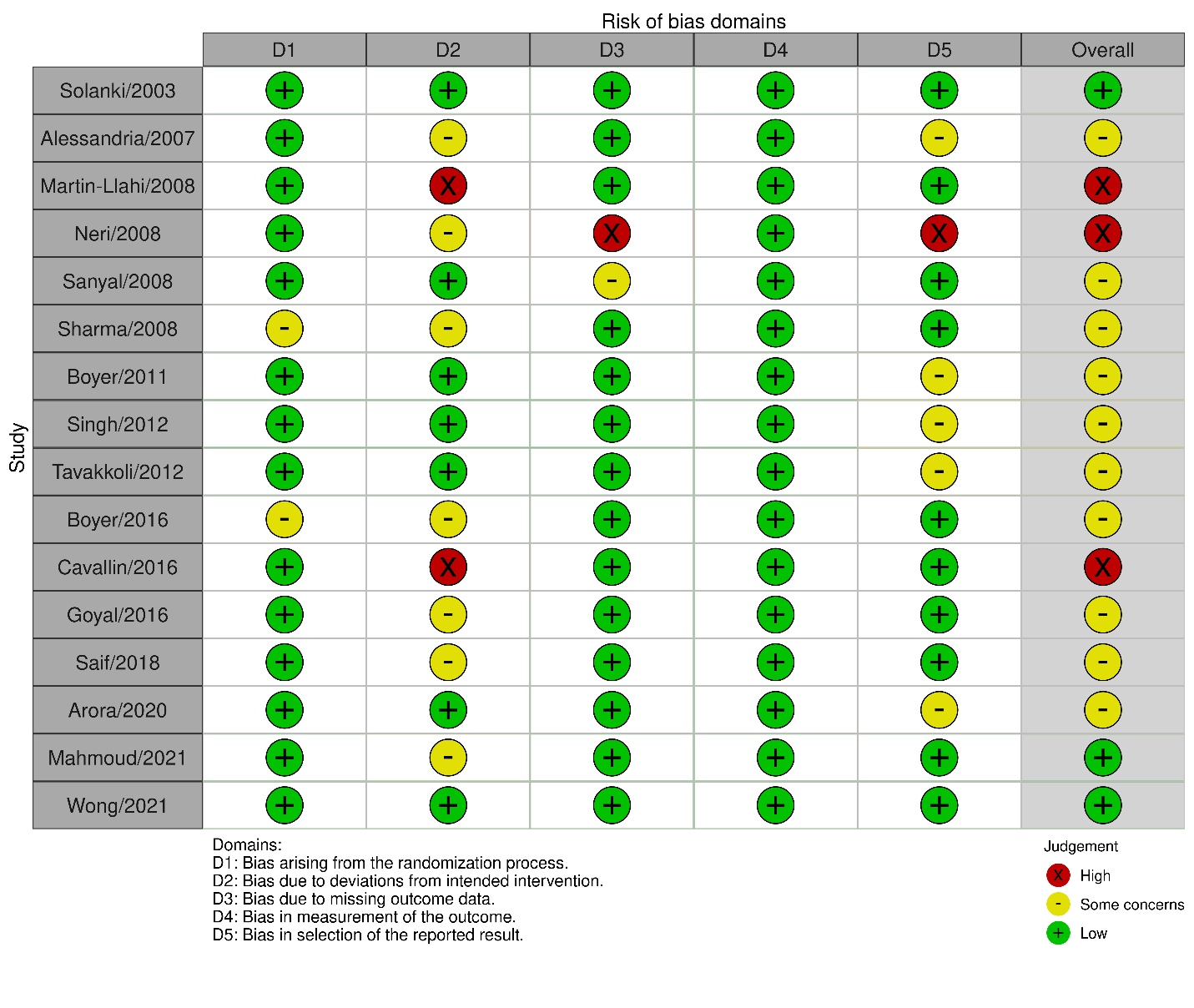


**Supplementary Figure 1** Risk of bias summary: Review authors’ judgments about each risk of bias item for the included studies.


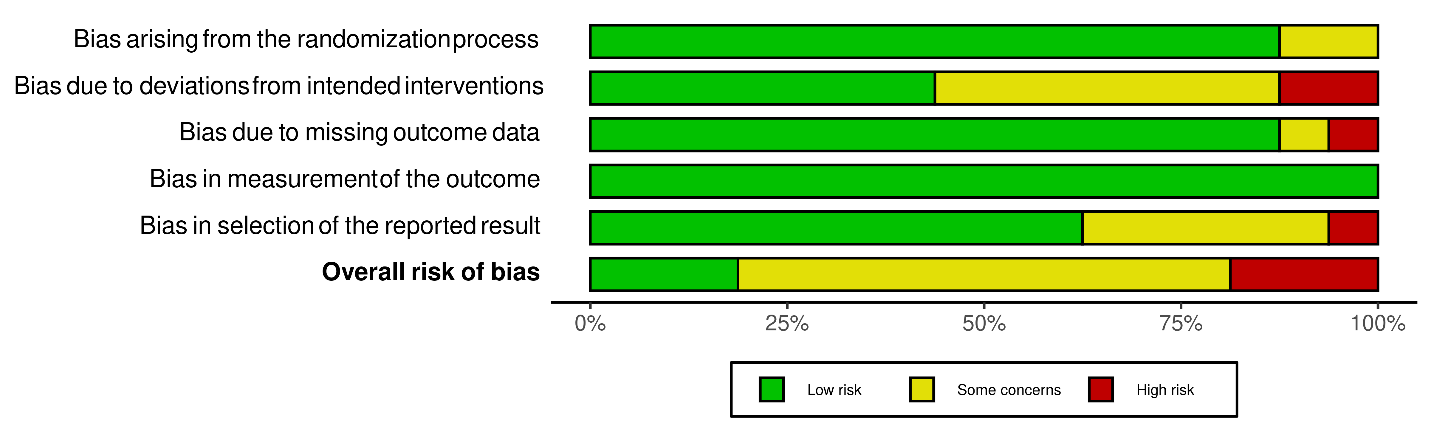


**Supplementary Figure 2** Risk of bias graph: Review authors’ judgments about each risk of bias item presented as percentages across all included studies.

**
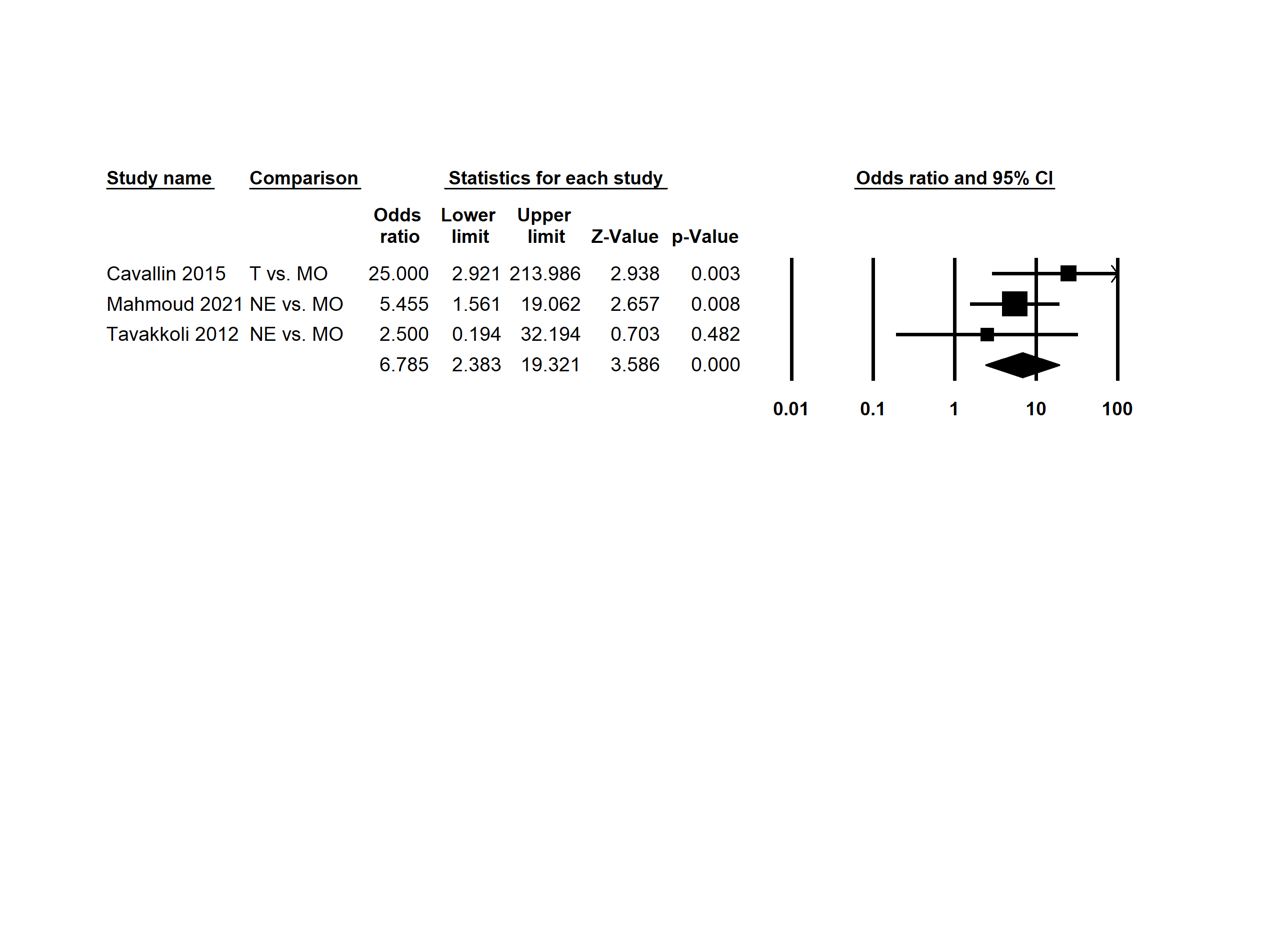
**

**Supplementary Figure 3A** Forrest plot comparing treatment with terlipressin or nor-epinephrine vs. midodrine and octreotide among patients with HRS-AKI on the pooled odds of reversal of hepatorenal syndrome. The bottom row and diamond sign represents pooled effect size with odds ratio with 95% confidence interval.

**
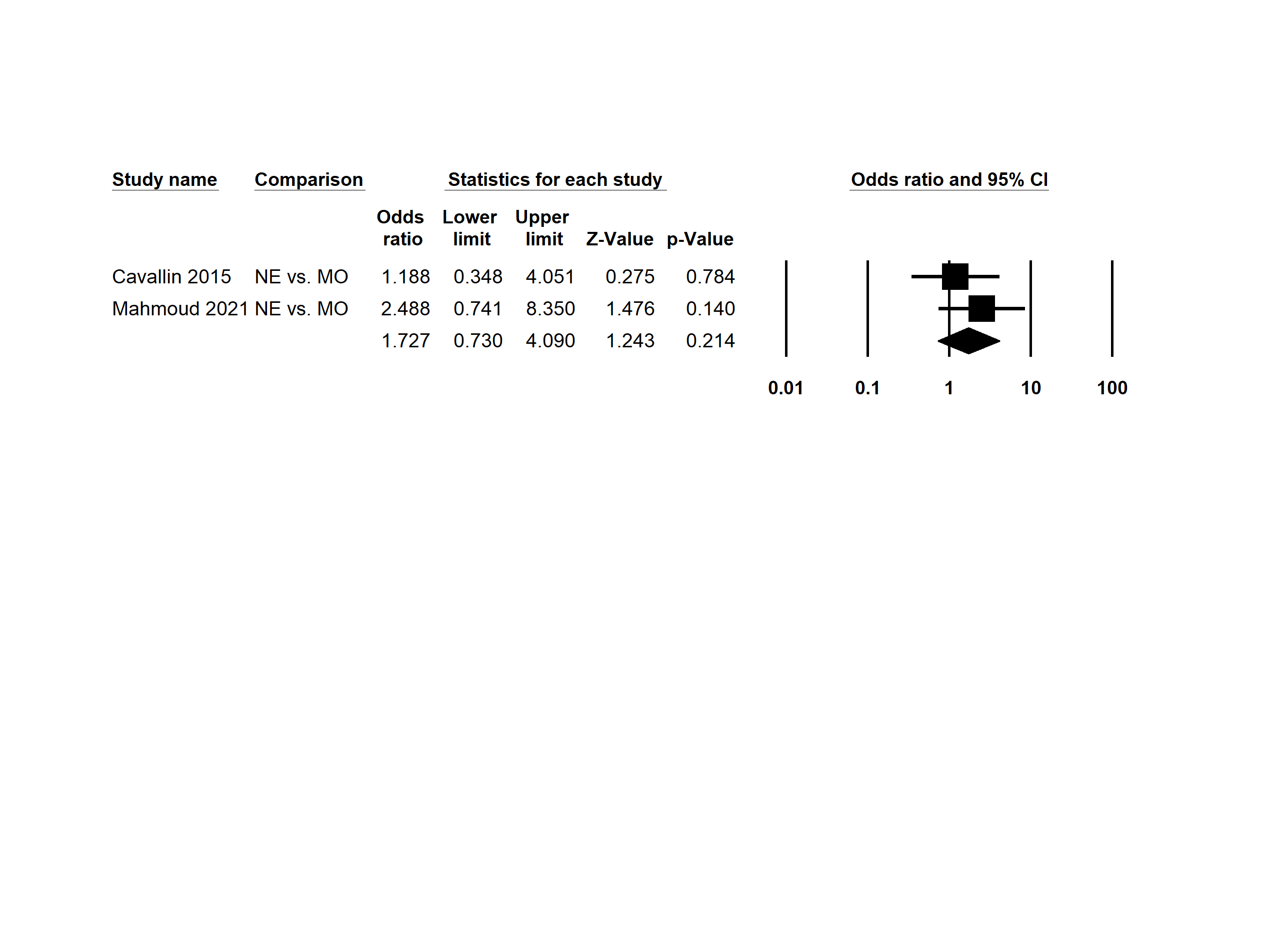
**

**Supplementary Figure 3B** Forrest plots comparing treatment with terlipressin or nor-epinephrine vs. midodrine and octreotide among patients with HRS-AKI on the pooled odds of liver transplant free patient survival at 30 days. The bottom row and diamond sign represents pooled effect size with odds ratio with 95% confidence interval.

**
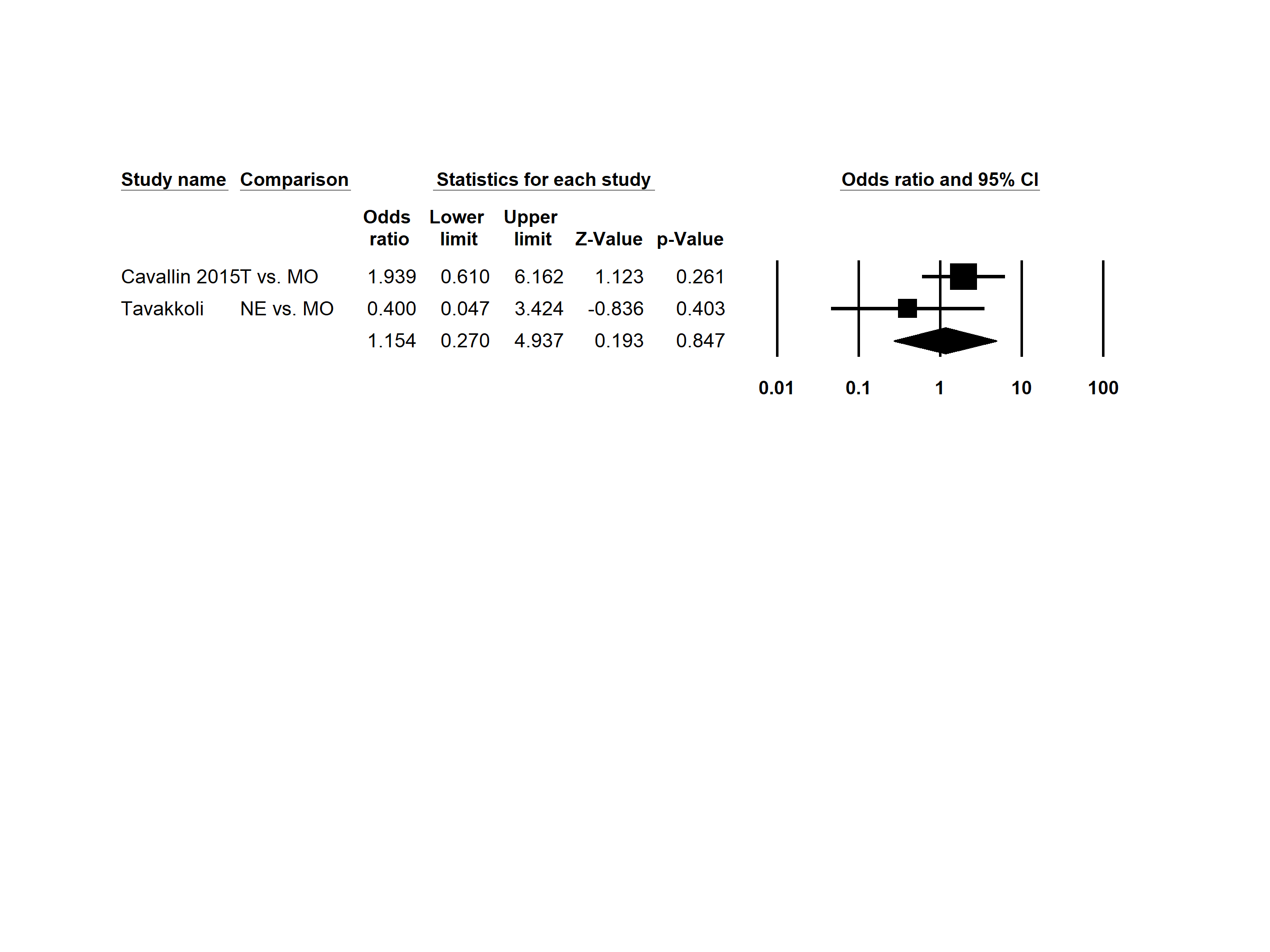
**

**Supplementary Figure 3C** Forrest plot comparing treatment with terlipressin or nor-epinephrine vs. midodrine and octreotide among patients with HRS-AKI on the pooled odds of liver transplant free patient survival at 90 days. The bottom row and diamond sign represents pooled effect size with odds ratio with 95% confidence interval.
